# Measurement of Thermal Conductivity and Thermal Diffusivity of Porcine and Bovine Kidney Tissues at Supraphysiological Temperatures up to 93 °C

**DOI:** 10.3390/s23156865

**Published:** 2023-08-02

**Authors:** Leonardo Bianchi, Silvia Fiorentini, Sara Gianella, Sofia Gianotti, Carolina Iadanza, Somayeh Asadi, Paola Saccomandi

**Affiliations:** Department of Mechanical Engineering, Politecnico di Milano, 20156 Milan, Italy; leonardo.bianchi@polimi.it (L.B.); silvia2.fiorentini@mail.polimi.it (S.F.); sara.gianella@mail.polimi.it (S.G.); sofia.gianotti@mail.polimi.it (S.G.); carolina.iadanza@mail.polimi.it (C.I.); somayehasadi786@gmail.com (S.A.)

**Keywords:** thermal properties measurement, thermal conductivity, thermal diffusivity, thermal measurements, kidney, thermal therapy modeling

## Abstract

This experimental study aimed to characterize the thermal properties of ex vivo porcine and bovine kidney tissues in steady-state heat transfer conditions in a wider thermal interval (23.2–92.8 °C) compared to previous investigations limited to 45 °C. Thermal properties, namely thermal conductivity (*k*) and thermal diffusivity (*α*), were measured in a temperature-controlled environment using a dual-needle probe connected to a commercial thermal property analyzer, using the transient hot-wire technique. The estimation of measurement uncertainty was performed along with the assessment of regression models describing the trend of measured quantities as a function of temperature to be used in simulations involving heat transfer in kidney tissue. A direct comparison of the thermal properties of the same tissue from two different species, i.e., porcine and bovine kidney tissues, with the same experimental transient hot-wire technique, was conducted to provide indications on the possible inter-species variabilities of *k* and *α* at different selected temperatures. Exponential fitting curves were selected to interpolate the measured values for both porcine and bovine kidney tissues, for both *k* and *α*. The results show that the *k* and *α* values of the tissues remained rather constant from room temperature up to the onset of water evaporation, and a more marked increase was observed afterward. Indeed, at the highest investigated temperatures, i.e., 90.0–92.8 °C, the average *k* values were subject to 1.2- and 1.3-fold increases, compared to their nominal values at room temperature, in porcine and bovine kidney tissue, respectively. Moreover, at 90.0–92.8 °C, 1.4- and 1.2-fold increases in the average values of *α*, compared to baseline values, were observed for porcine and bovine kidney tissue, respectively. No statistically significant differences were found between the thermal properties of porcine and bovine kidney tissues at the same selected tissue temperatures despite their anatomical and structural differences. The provided quantitative values and best-fit regression models can be used to enhance the accuracy of the prediction capability of numerical models of thermal therapies. Furthermore, this study may provide insights into the refinement of protocols for the realization of tissue-mimicking phantoms and the choice of tissue models for bioheat transfer studies in experimental laboratories.

## 1. Introduction

The wide-spectrum characterization of the physical properties of biological tissue has always elicited the interest of the biomedical community. Indeed, quantitative information on tissue characteristics is needed for the improvement and accurate design of interventional procedures, the validation of medical tools, and the realization of tissue-mimicking materials to be employed for medical training and the refinement of therapeutic treatments [1,2,3,4]. Among the different thermo-electro-mechanical characteristics of tissues, tissue thermal properties underlie the mechanisms of heat transfer within biological media [5]. Hence, their accurate estimation is also favorable in view of the increased effectiveness and safety of thermal-energy-based therapies for the treatment of various diseases [6,7,8,9,10]. The thermal properties regulate the processes of conduction, storage, and release of thermal energy within the tissue [5]. The thermal conductivity (*k*) of tissue is related to the ability of the biological media to conduct thermal energy. Moreover, the thermal diffusivity (*α*) of a tissue quantitatively represents its capability to transfer heat in relation to storing thermal energy [9,11,12].

In order to perform a thorough characterization of the tissue’s thermal features and provide valuable information for the optimization of the therapeutic procedures involving temperature changes to treat a disease (such as thermal therapies), it is vital to measure the tissue’s thermal properties as a function of temperature [9,12]. Indeed, different phenomena occur in biological media when the tissue temperature changes from body temperature up to supraphysiological temperatures [9,13,14,15]. Variations in the tissue temperature from the baseline conditions up to 41 °C have been shown to increment the diffusion of ions across the cellular membranes, as well as vasodilatation and blood flow [16]. Furthermore, at higher thermal intervals, such as in hyperthermia temperature ranges, i.e., between 42 °C and 45–48 °C, inactivation of vital enzymes alongside unfolding and aggregation of proteins and hampered repair to DNA damage may occur [17]. Since the application of hyperthermic temperatures may lead to irreversible thermal damage in tissue exposed to these temperatures for 30–60 min [18], hyperthermia is employed as a cancer therapy modality [18,19,20]. Delivering the proper thermal dose to target tissues is essential for the efficacy of hyperthermia therapy. Therefore, monitoring the thermal characteristics of tissue and comprehending how they vary with temperature can benefit the development of safer and more precise hyperthermia procedures [15,21]. At higher temperatures (50–60 °C), the time required to attain the definitive tissue thermal injury is reduced. Moreover, at temperatures > 60 °C, thermal damage can be achieved due to almost immediate protein denaturation leading to coagulative necrosis [18]. Thermo-ablative procedures, such as radiofrequency [22,23], microwave [24,25], laser ablation [26,27,28], and high-intensity focused ultrasound [29], operate at these temperatures and above, to induce coagulative necrosis in tissue for tumor treatment [30]. Moreover, at temperatures >80–90 °C, phenomena such as drying and water vaporization occur [31,32].

Considering the high thermal gradients and temperature excursions to which the tissue may be exposed during thermal therapies [32], the temperature dependence of the thermal properties of biological tissue must be assessed in a wide thermal interval, ranging from nominal up to supraphysiological (i.e., hyperthermic and ablative) temperatures [9,12,21,33]. Different techniques have been adopted to characterize the thermal properties of biological media as a function of tissue temperature. Self-heated thermistor probes have been utilized to measure the *k* and *α* of various human and animal tissues and assess their temperature dependence [34,35,36]. Moreover, in more recent years, the dual-needle technique based on the transient hot-wire method has been employed for the estimation of the thermal properties of porcine, bovine and ovine liver; muscle; porcine brain; pancreas; lung and heart [7,12,33,37,38,39].

However, an analysis of the literature in the field reveals that a limited number of investigations have been carried out to determine thermal properties as functions of the temperature (from room temperature up to hyperthermia and ablative temperatures) of tissues that are a target of thermo-ablative procedures [9,21], such as kidney tissues [35,40].

The kidney is a vital organ that is involved in numerous life-sustaining functions, including blood filtering, controlling fluid and electrolyte balance, and secreting hormones for the regulation of blood pressure [41]. Despite its significance, a number of conditions and diseases can affect the kidney, e.g., renal cell carcinoma [42], among others. In this case, thermal ablation procedures represent a valid treatment option for patients who are not eligible for surgical procedures [42,43]. In addition to tumor thermal ablation, other treatment modalities, such as catheter-based renal denervation, involve the use of thermal energy for treatment purposes [44]. Renal denervation is a minimally invasive treatment for patients with resistant hypertension, and it involves using a catheter to apply thermal energy to the nerves in the renal arteries, decreasing nerve activity, and hence blood pressure [44]. The success of these interventions depends on the accurate delivery of heat to the target tissue and biological structures, which can be optimized by characterizing the temperature dependence of the thermal properties of kidney tissue.

Among the few experimental trials performed for the measurement of the thermal properties of kidney tissue as a function of temperature, one of the first and more comprehensive investigations is the study conducted by Valvano et al. [35]. In their study, the *k* and *α* of different biological tissues were measured using self-heating thermistor probes. In particular, the thermal properties of an ex vivo porcine renal cortex, rabbit kidney, human renal pelvis, human renal medulla, and human renal cortex were measured at 3 °C, 10 °C, 17 °C, 23 °C, 30 °C, 37 °C and 45 °C [35]. In recent years, the dual-needle technique was employed by Silva et al. to evaluate the thermal properties of ex vivo ovine kidney tissue from nominal conditions at room temperature up to 95–96 °C [40]. The measurement results show variations in thermal properties at temperatures approaching the onset of water vaporization. Concerning non-invasive measurement techniques, Dragonu et al. presented a method for estimating the *α* of ex vivo perfused porcine kidney tissue undergoing high-intensity focused ultrasound treatments monitored via volumetric magnetic resonance imaging (MRI) thermometry [45]. The value of *α* was estimated by the evaluation of the spatial spread of the temperature over time during the cooling phase. Additionally, Cornelis et al. quantitatively evaluated the *α* of in vivo porcine kidneys during MRI-guided, high-intensity focused ultrasound [46]. The bioheat transfer model was utilized to analyze temperature results and quantitatively estimate the *α* of renal tissue. The reported *α* was equal to 0.23 ± 0.11 mm^2^ s^−1^ and expressed as mean ± standard deviation. In the previously described studies [35,40], *k* and *α* values were reported for kidney tissues of different animals. Valvano et al. reported the inter-species dependence as one of the major causes of uncertainty in the estimation of these properties [35]; therefore, an analysis of this factor using the same measurement technique is necessary.

In this study, prompted by the necessity to extend the temperature interval in which the thermal properties of renal tissue are investigated, we performed the first ever devised characterization of the *k* and *α* of ex vivo porcine and bovine kidney tissues from room temperature up to supraphysiological and ablative temperatures (i.e., 90–93 °C). The choice of characterizing the properties of porcine and bovine tissues is dictated by the fact that these tissues are often advocated as experimental models in research laboratories for the study, testing, and improvement of thermal-energy-based treatment modalities and devices [23,47]. The measurement of thermal properties was performed in steady-state conditions in a temperature-monitored environment by means of the transfer hot-wire technique employing a dual-needle probe. The measurement accuracy of the system was validated by our research group in previous studies [12,33,39], and its accurate reading capability has also been assessed in the present work by testing the measurement instrument on a polymeric standard material. The quantitative values of the measured thermal properties of biological tissues are presented, along with the estimation of the measurement uncertainty, the best-fit regression models interpolating the experimental data, and the analysis of the residual values. The comparison between the thermal properties of porcine and bovine kidney tissues was also performed to provide indications on the possible inter-species variabilities of these properties at different selected temperatures from room temperature up to body and supraphysiological temperatures. The overall aim is to provide data and regression models that can be used in mathematical models of thermal therapies [48,49,50,51] and for the realization of materials resembling tissue properties and behavior [1,3,52].

## 2. Experimental Procedure and Methods for Data Analysis

### 2.1. Experimental Setup

**Figure 1:**
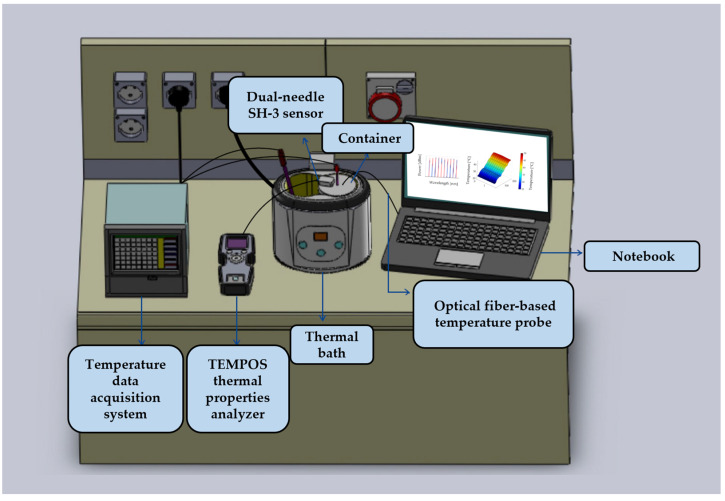
Experimental setup, consisting of a temperature monitoring module, the TEMPOS commercial analyzer with the dual-needle SH-3 sensor, a water thermal bath, the kidney tissue sample in the metallic container, thermocouples, and an optical fiber temperature probe (embedding 10 FBG sensors) employed in order to monitor the temperature, and a notebook, connected to the optical interrogation unit, to collect data from the optical fiber probe.

**Figure 2:**
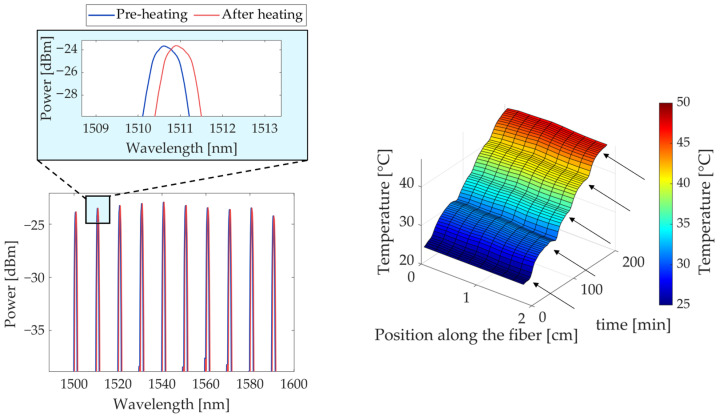
A fiber optic temperature probe was introduced into the kidney tissue to monitor the spatial variations of the temperature of the sample during heating. The reflection spectra showing the 10 peak Bragg reflections before and after heating are shown on the left. On the right, a representative thermal map across the tissue depth and time is depicted. The graph refers to an experimental trial on porcine kidney tissue and shows the thermal values from room up to hyperthermic temperatures. The arrows indicate when the measurement of the thermal properties was performed, i.e., in steady-state heat transfer conditions when the tissue sample reached thermal equilibrium.

The experimental setup is shown in Figure 1, and it consists of the following parts. A water thermal bath was employed to raise the temperature of the sample up to the desired temperature values and then maintain the tissue temperature at the selected value while measuring its thermal properties. A thermally conductive metallic container was filled with the tissue sample in order to protect the tissue from direct contact with water (details are reported in Section 2.2). Two k-type thermocouples (0.1 °C accuracy, associated with a temperature monitoring module, Yokogawa FX1000 Paperless Recorder) were used to monitor the heating procedure in the tissue and the water bath, respectively. Furthermore, a fiber-optic-based temperature probe was introduced into the kidney tissue to monitor the spatial variations of the temperature of the sample during heating, as shown in Figure 2. This probe consists of 10 fiber Bragg grating (FBG) sensors. FBG sensors allow us to monitor the temperature variation of the medium in which they are located by detecting, through an optical interrogation unit, the change in the light reflected by the sensing elements (also referred to as gratings). Once interrogated, each grating, photo-inscribed in the core of the optical fiber, reflects a narrow-band region of the spectrum. This spectral component is centered at the so-called Bragg wavelength, which is characteristic of each grating and depends upon the effective refractive index of the core mode field and the spatial grating period [26,27,53,54,55]. Following a change in the temperature of the medium with which the FBG sensors are in contact, the thermal expansion of the fiber material and effective refractive index changes result in a shift in the Bragg wavelengths associated with the different gratings. Hence, tracking the shifts of the Bragg wavelength peaks of the reflected spectra allows for the indirect measurement of the thermal changes within the material under study, in correspondence with the sensor locations. The 10 FBGs each have a 1 mm sensing length and edge-to-edge distance of 1 mm; hence, the total sensing length is 19 mm (FiSens GmbH, Braunschweig, Germany). The starting temperature of the experiment was around 23 °C. An optical spectrum interrogator (Micron Optics si255, Atlanta, GA, USA, 1 pm accuracy corresponding to 0.1 °C) was used to collect the optical output of the FBG sensors. The TEMPOS thermal properties analyzer (TEMPOS, Meter Group, Inc., Pullman, WA, USA, accuracy: 10%) equipped with the SH-3 dual-needle sensor (measurement range: −50–150 °C) was used to measure the thermal properties of the kidneys. The needles of this measurement system are 30 mm long, 1.3 mm in diameter, and 6 mm apart. These dimensions allow for the suitable insertion of the dual needle into the tissue samples.

### 2.2. Tissue Preparation

**Figure 3:**
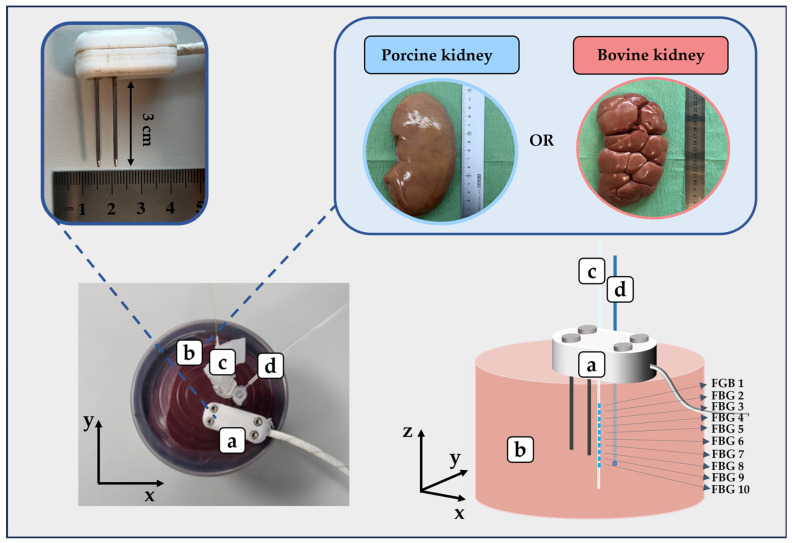
The tissue sample was located in the metallic container with a silicone lid. Then, the probes were inserted into the sample and kept in place during the measurement of thermal properties. On the left, the picture of the metallic container is reported, showing (a) the dual-needle SH-3 probe, with a focus on the two needles (3 cm in length) used for the measurements; (b) the tissue samples, i.e., either ex vivo porcine or bovine kidney tissue; (c) the optical-fiber-based temperature probe; and (d) the k-type thermocouple. On the right, the three-dimensional schematic representation of (a) the dual-needle probe, (b) the kidney tissue sample, (c) the optical-fiber-based temperature probe embedding an array of 10 fiber Bragg grating (FBG) sensors, and (d) the k-type thermocouple is shown.

**Figure 4:**
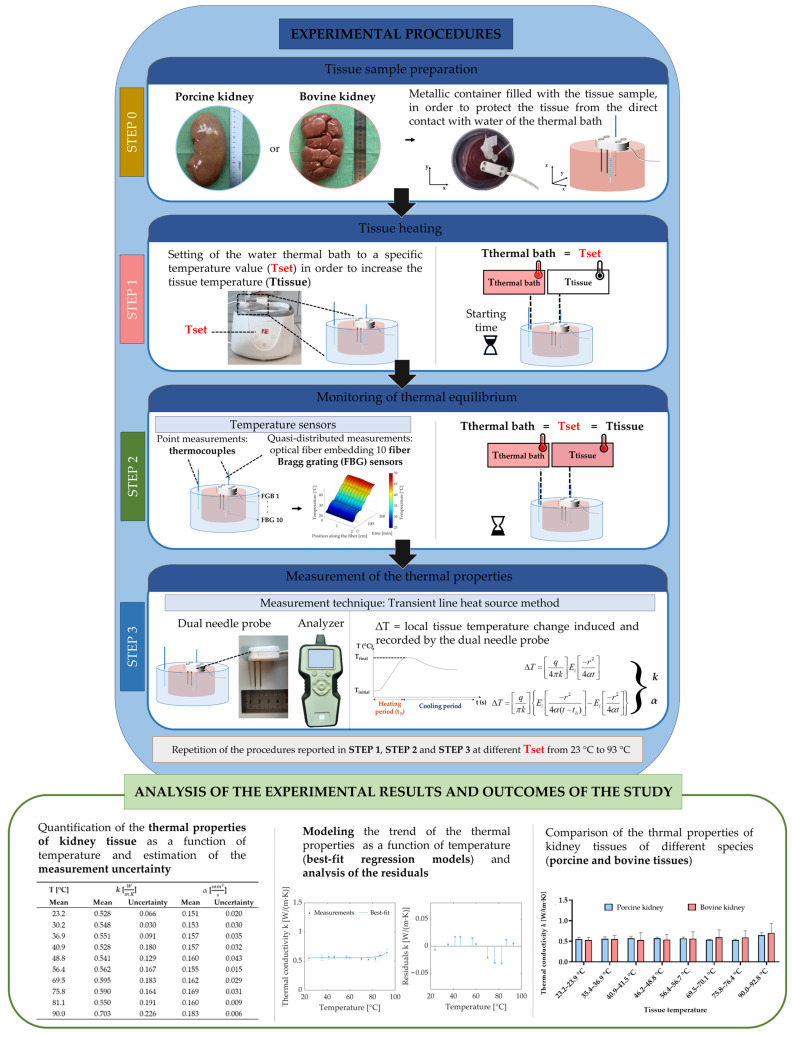
Flowchart outlining the experimental steps and procedures, the performed analysis and the expected outcomes of the study.

All the experiments were conducted in porcine and bovine kidney samples obtained from a local butcher, i.e., six porcine and six bovine specimens. The tissues were freshly excised, refrigerated, and then allowed to reach room temperature before proceeding with the experiments. The experiments were performed with the aim of preserving the structure of the kidneys and penetrating both the cortex and medulla with the dual-needle probe [38]. A metallic container was covered with a silicone lid avoiding air bubbles that may affect measurements. The used lid was drilled with four holes, with the aim of inserting the SH-3 dual-needle sensor used to measure the thermal properties of the tissue. Additionally, thermometers were used to monitor tissue temperature during the heating procedure, i.e., one thermocouple and a fiber-optic-based temperature probe (Figure 3). The thermocouple and fiber optic probe were inserted into the tissue using needles, which were placed close to the SH-3 sensor. Indeed, the TEMPOS system constituted by the SH-3 probe and the thermal analyzer is itself capable of resolving temperature to ±0.001 °C. The decision to employ two additional temperature sensors close to the SH-3 dual-needle probe was made to ensure the absence of thermal gradients in the region of the tissue close to the dual-needle probe during the measurement of thermal properties. It has been proved that a minimum of 4 mm of material must be parallel to the dual-needle probe in all directions to guarantee accurate measurements [8]. Hence, we wanted to verify the steady-state heat transfer conditions during measurements of *k* and *α*, especially in the volume of tissue closely surrounding the SH-3 sensor. The position of all the probes inserted in the tissue was kept still using strips of parafilm and tape to avoid displacement of the sensors during measurements. An additional thermocouple was inserted into the water of the thermal bath. Figure 4 reports the flowchart outlining the experimental procedures and analysis performed in the present study.

### 2.3. Heating Protocol and Measurement of the Thermal Properties

The container was placed into the water thermal bath to reach the desired temperature, which was kept constant during the measurement of thermal properties. The thermal bath was set to specific temperatures for each sample, and measurements of the thermal properties were performed in steady-state heat transfer conditions, i.e., only when the sample reached thermal equilibrium. The temperature range of interest set in the thermal bath was ~22 °C to >90 °C, and was divided into 10 steps for porcine kidneys and 9 steps for bovine kidneys. The TEMPOS thermal properties analyzer equipped with an SH-3 dual-needle sensor was used to measure *k* and *α*, according to the transient line heat source method [7,9,12,33,37,38,39]. The adopted measurement instrument based on the transient line heat source method is characterized by several advantages in comparison with standard steady-state techniques for the measurement of thermal properties [56,57]. Indeed, the employed system ensures the minimization of the thermally induced water movement in specimens by reducing the time taken for tissue heating periods and measurements. Furthermore, the heating of the tissue is constrained, resulting in low heating rates and reducing free convection and water vaporization. The mentioned features are supported by highly resolved temperature measurements. Indeed, as previously mentioned, the TEMPOS system constituted by the SH-3 probe and thermal analyzer is capable of resolving temperature to ±0.001 °C [58]. In the following, the explanation of the measurement mechanism is provided. The measurement process related to the SH-3 dual-needle sensor is based on the application of a certain quantity of heat to the heating needle for 30 s, as current passes through the heater. The other needle monitors the subsequent temperature variation for the following 90 s. Typically, the temperature rise in tissue due to the heat provided by the heating needle is <1 °C. Each measurement requires an overall time of 2 min. Then, the initial temperature value measured at the onset of the heating period and the temperature drift are subtracted from the temperature registered by the monitoring needle in order to quantify the temperature change over time. The values of *k* and *α* are derived by fitting the resulting data to Equations (1) and (2) using a least square method. This method minimizes the sum of the squares of the differences between the mathematical model of heat transfer implemented in the software of the thermal analyzer and the measured values:

(1) $$\Delta T=\left[\frac{q}{4\cdot \pi \cdot k}\right]E_{i}\left[\frac{-r^{2}}{4\cdot \alpha \cdot t}\right]\;\;t\leq t_{h}$$

(2) $$\Delta T=\left(\frac{q}{\pi \cdot k}\right)\left\{E_{i}\left[\frac{-r^{2}}{4\cdot \alpha \cdot \left(t-t_{h}\right)}\right]-E_{i}\left[\frac{-r^{2}}{4\cdot \alpha \cdot t}\right]\right\}\;t>t_{h}$$

where ∆*T* refers to the temperature increment at the measuring needle (typically around <1 °C), *q* concerns the heat input at the heating needle [W/m], *k* is thermal conductivity [W/(m·K)], *r* is the distance between the two needles (6 mm), *α* is thermal diffusivity [mm^2^/s], *t* is time [s], *t_h_* is heating time [s] (30 s), and $E_{i}$(−*x*) is the exponential integral, approximated using polynomials: in particular, for small arguments, it is approximated as the sum between the Euler–Mascheroni constant (γ ≈ 0.5772) and the natural logarithm of the argument, *ln*(*x*) [33,37,59].

### 2.4. Measurement Uncertainty Evaluation

For each thermal parameter, the mean value $\bar{y}$ and the corresponding expanded measurement uncertainty (*EU*) were calculated. Each experimental trial concerning the measurement of the thermal properties from room to ablative temperatures was repeated three times for each tissue type, i.e., three experimental trials from room to ablative temperatures were performed for the measurement of the thermal properties of porcine kidney. Three experimental trials from baseline to ablative temperatures were also conducted for the measurement of the thermal properties of bovine tissue.

(3) $$y=\bar{y}\pm EU$$

The *EU* is defined by *s*, given in Equation (4), multiplied by the coverage factor *k_f_*. This coverage factor is obtained considering a confidence level of 95% for a Student’s *t*-distribution. Thus, having 3 measurements (2 degrees of freedom), *k_f_* = 4.30 [60].

(4) $$s=\sqrt{\frac{\sum_{i=1}^{n}{\;\left(y_{i}-\bar{y}\right)}^{2}}{n\left(n-1\right)}}$$

(5) $$EU={s\text{·}k}_{f}$$

where *n* is the number of experiments for each temperature (*n* = 3), *y* is the measured thermal property and $\bar{y}$ is the arithmetic mean.

### 2.5. Thermal Property Modeling

In order to obtain the trend of the studied properties as a function of the temperature, the best-fit model was obtained for each thermal parameter. Concerning the modeling of thermal properties, the best-fit model for *k* and *α* is represented by an exponential curve as follows:

(6) $$y(T)=a+b\cdot e^{\left(cT\right)}$$

The best-fit models for the collected data and the best parameters of the equation estimated using the least squares method were obtained using MATLAB^®^ (Mathworks, Natick, MA, USA). Finally, the coefficient of determination ($R^{2}$) was estimated, and the analysis of residuals was performed.

### 2.6. Statistical Analysis

The differences between the values of the thermal properties of porcine and bovine kidney tissue, at the same tissue temperatures, were analyzed by applying a two-tailed unpaired Student’s *t*-test. Differences with *p*-values < 0.05 were considered statistically significant.

## 3. Results

### 3.1. Measurement System Validation

Prior to proceeding with actual measurements of thermal properties in ex vivo biological tissues, measurements were conducted on a reference material provided by the manufacturer (white plastic Delrin^®^ cylinder in polyoxymethylene) to ensure the measurement system was accurate and functioned correctly without any flaws. At room temperature, i.e., 22.3–26.0 °C, the results of our measurements were *k* = 0.379 W/(m∙K) and *α* = 0.189 mm^2^/s, while the values reported on the Certificate of Quality Assurance are *k* = 0.384 W/(m∙K) and *α* = 0.189 mm^2^/s. The difference of <1.5% between our measurement results on the standard material and the values reported by the manufacturer indicated the accurate reading capability of our system [61].

In the following sections, the results of the measured *k* and *α* of porcine and bovine kidney tissues are reported for the investigated thermal ranges.

### 3.2. Thermal Properties of Porcine Kidney

The thermal properties of ex vivo porcine kidney tissue were measured in steady-state heat transfer conditions at different tissue temperatures, i.e., from nominal conditions at room temperature (23.9 °C) to approximate body temperature (35.4 °C), and up to hyperthermia and ablative temperatures (41.5 °C, 46.2 °C, 56.7 °C, 60.0 °C, 70.1 °C, 76.4 °C, 82.3 °C, 86.6 °C, 92.8 °C). The attained results, in terms of the average values of *k* and *α*, and the associated measurement uncertainty for the investigated tissue temperatures (estimated with a 95% confidence level) are reported in Table 1.

**Figure 5:**
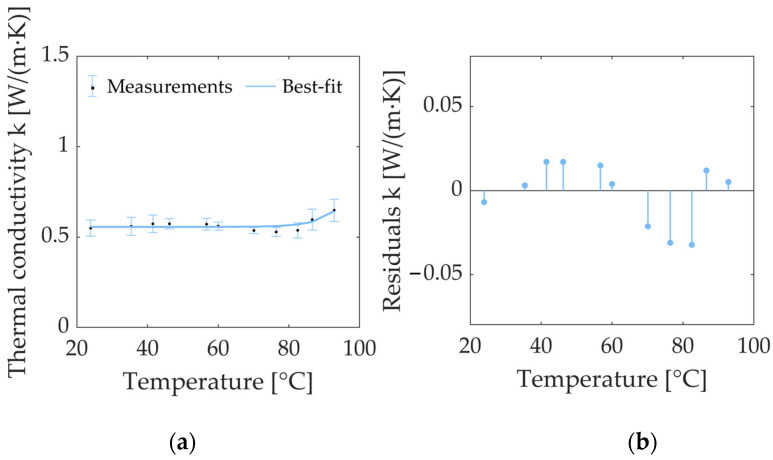
(**a**) Thermal conductivity *k* of ex vivo porcine kidney as a function of temperature: the measured values (average values) and measurement uncertainty estimated with a 95% confidence level are reported together with the approximate values of the best-fit curve. (**b**) The plot of residuals of thermal conductivity *k* of porcine kidney as a function of temperature. The *x*-axis is defined by the temperature value measured using the SH-3 dual-needle sensor.

**Figure 6:**
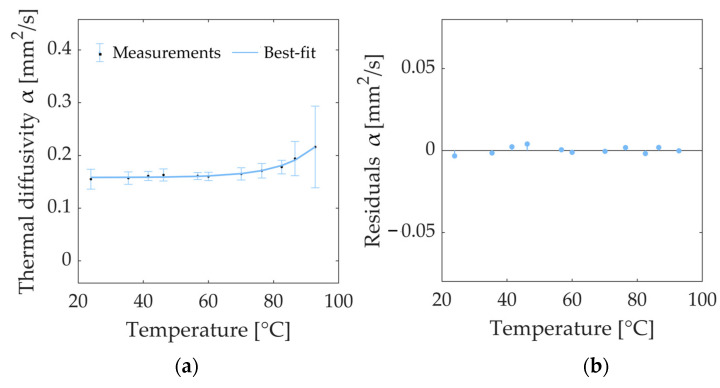
(**a**) Thermal diffusivity *α* of ex vivo porcine kidney as a function of temperature: average values (dots) and measurement uncertainty estimated with a 95% confidence level are shown along with the best-fit curve interpolating the experimental data (solid line). (**b**) Plot of residuals of thermal diffusivity *α* of porcine kidney tissue as a function of temperature. The *x*-axis is defined by the temperature measured using the SH-3 dual-needle sensor.

In order to visualize the trend of the thermal properties with increasing tissue temperatures, the data of *k* and *α*, reported in Table 1, are, respectively plotted in graphs as a function of temperature in Figure 5 and Figure 6.

Concerning the *k* of porcine kidney tissue, its nominal value at room temperature was equal to 0.549 W/(m·K). A gradual increase from 23.9 °C to 46.2 °C, followed by a slight decline from 60.0 °C to 82.3 °C, can be observed. Finally, an exponential growth up to 92.8 °C was registered, with a value of 0.648 W/(m·K) at 92.8 °C. Comparing the value of *k* at the initial value and last value of the temperature interval (23.9 °C and 92.8 °C, respectively) an overall increase of 18% was observed at 92.8 °C with respect to the baseline value measured at room temperature.

Figure 5a also depicts the best-fit model for the accurate approximation of the trend of the experimentally investigated *k* values, which are represented by an exponential curve defined using Equation (6). The regression coefficients are reported in Table 2, along with the R^2^. The results of the residuals analysis are reported in Figure 5b. The residuals appear distributed around the y = 0 value, and their values at different temperatures are <7% of the measured property values. The best-fit curve slightly underestimates the values in the range between 35.4 °C and 60.0 °C, while it overestimates them in the 70.1–82.3 °C range. Lastly, a slight underestimation is notable at temperatures higher than 82.3 °C.

Concerning the measurement of *α* at different temperatures, *α* remained almost constant from room temperature up to 60.0 °C, showing values in the interval of 23.9–60 °C, ranging from a minimum of 0.155 mm^2^/s (observed at 23.9 °C) to a maximum of 0.163 mm^2^/s (at 46.2 °C). After 60.0 °C, the value *α* increased with temperature, reaching values of 0.178 mm^2^/s at 82.3 °C, 0.194 mm^2^/s at 86.6 °C, and 0.216 mm^2^/s at 92.8 °C, corresponding to increases, compared to the baseline values, of 14%, 25%, and 39%, respectively. Figure 6 shows the values of *α* at the different temperatures along with the best-fit curve interpolating the experimental measurements. The trend of *α* as a function of tissue temperature was modeled using an exponential curve (Equation (6)), whose regression coefficients are reported in Table 2. Regarding the analysis of the residuals, the residual plot is depicted in Figure 6b. The residuals are distributed around the value y = 0, and their values are lower than 3% of the property values. According to the residual plot, the chosen exponential model slightly overestimates the values in the 23.9–35.4 °C thermal range and at 82.3 °C, whilst it slightly underestimates the values at 41.5–46.2 °C, 76.4 °C, and 86.6 °C.

### 3.3. Thermal Properties of Bovine Kidney

The same experimental setup employed for the evaluation of the temperature dependence of the thermal properties of porcine kidney tissue was utilized for the measurement of *k* and *α* of ex vivo bovine tissues as a function of temperature from 23.2 °C up to 90.0 °C. Table 3 reports the average values of the thermal properties at the different selected tissue temperatures (23.2 °C, 30.2 °C, 36.9 °C, 40.9 °C, 48.8 °C, 56.4 °C, 69.5 °C, 75.8 °C, 81.1 °C, and 90.0 °C) along with the estimated measurement uncertainty.

**Figure 7:**
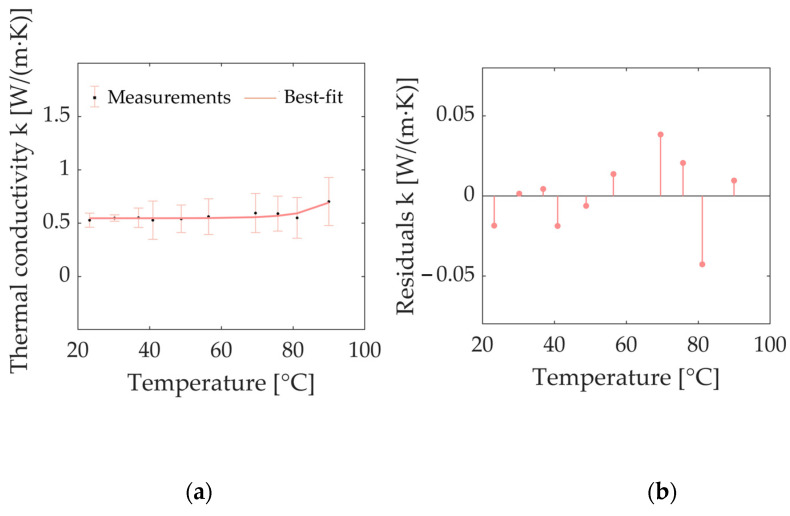
(**a**) Thermal conductivity *k* of bovine kidney tissue as a function of temperature: measured values (average values and associated measurement uncertainty estimated with a 95% confidence level) together with the best-fit curve approximating the experimental values (solid line) are observable. (**b**) Plot of residuals of thermal conductivity *k* of bovine kidney tissue as a function of temperature. The *x*-axis is defined by the temperature measured using an SH-3 dual-needle sensor.

Figure 7a depicts the trend of the experimental data for the *k* of bovine kidney tissue, attained using the dual-needle technique. As is observed, the value of *k* exponentially increased when temperatures of 90 °C were reached. At nominal conditions, i.e., room temperature, *k* was equal to 0.528 W/(m·K); at body temperature (i.e., 36.9 °C), the average value of *k* was 0.551 W/(m·K). At the maximum investigated temperature value, i.e., 90.0 °C, *k* reached a value of 0.703 W/(m·K), showing increases of 33% and 28% compared to the baseline values at room and body temperatures, respectively. In Figure 7a, the best-fit curve interpolating the measured values of *k* is also shown. The exponential model (Equation (6)), similar to what was observed for the *k* of porcine kidney tissue, can be considered an accurate approximation of measured thermal property values at the different temperatures. Table 4 reports the regression coefficients of the exponential fitting curve and the *R^2^*, which is equal to 0.805. The analysis of the residuals over temperature (Figure 7b) shows that the residuals are distributed around the value of y = 0. Moreover, their values are <8% of the measured property values at all the investigated temperatures. From Figure 7b, it is possible to observe an underestimation of the values between 69.5 °C and 75.8 °C by the mathematical model chosen to approximate these experimental values and, conversely, an overestimation of the values at 81.3 °C.

**Figure 8:**
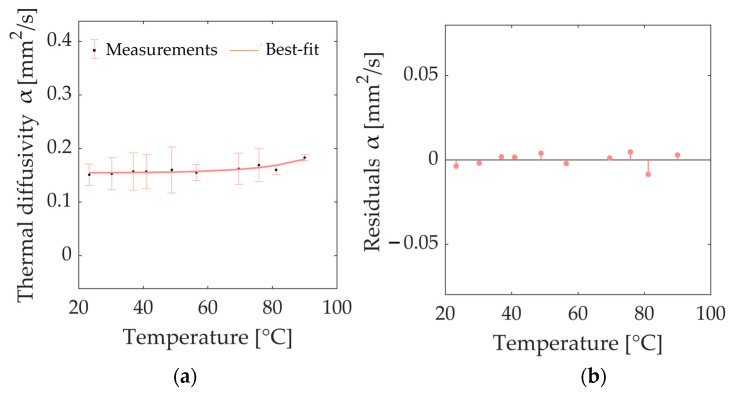
(**a**) Thermal diffusivity *α* of bovine kidney tissue as a function of temperature: average values and associated measurement uncertainty (estimated with a 95% confidence level) are depicted along with the best-fit curve interpolating the experimental values. (**b**) Plot of residuals of thermal diffusivity *α* of bovine kidney tissue as a function of temperature. The *x*-axis is defined by the temperature measured using the SH-3 dual-needle sensor.

Concerning the characterization of the *α* of bovine kidney tissue, from Table 3, it is possible to notice a baseline value at room temperature equal to 0.151 mm^2^/s. A value of 0.157 mm^2^/s was measured at body temperature, and a similar value (0.155 mm^2^/s) was registered at 56.4 °C. Moreover, an increment in the tissue temperature up to 90.0 °C led to an increase in *α* of 21% compared to the nominal value at ambient temperature. The exponential model, for which the regression coefficients are shown in Table 4, was selected to accurately approximate the trend of the measured *α* values (Figure 8a) and describe the temperature dependence of the *α* of bovine kidney tissue (*R^2^* equal to 0.809). Figure 8b reports the analysis of the residuals, which appear distributed around the y = 0 value and characterized by values lower than 6% of the measured property values for all investigated temperatures. As indicated by the residuals plot (Figure 8b), the chosen best-fit model slightly underestimates the values at 48.8 °C and 75.8 °C, whereas an overestimation is observed at 81.1 °C.

### 3.4. Comparison of the Thermal Properties of Porcine and Bovine Kidney Tissues

**Figure 9:**
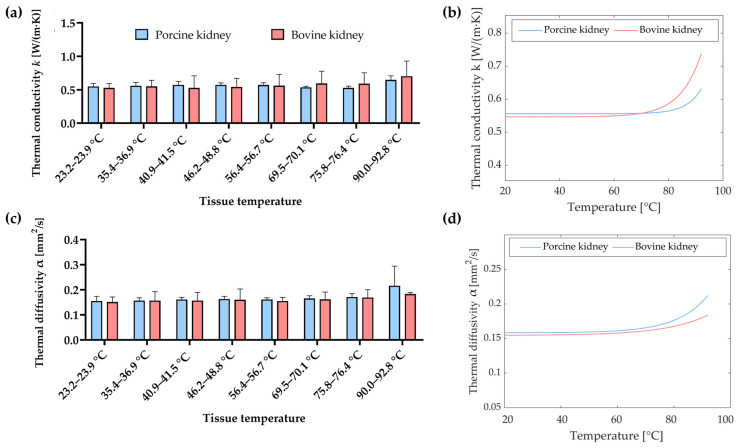
Comparison of the thermal properties of porcine and bovine kidney tissues: (**a**) Bar plot reporting the average values of *k* attained at temperatures of 23.2–23.9 °C, 35.4–36.9 °C, 40.9–41.5 °C, 46.2–48.8 °C, 56.4–56.7 °C, 69.5–70.1 °C, 75.8–76.4 °C, and 90.0–92.8 °C for both porcine and bovine samples, along with the associated measurement uncertainty (estimated with a 95% confidence level). (**b**) Best-fit regression curves interpolating the experimental *k* data for both porcine and bovine kidney samples. (**c**) Bar plot reporting the average values of *α* obtained for both porcine and bovine samples at the different selected temperatures, along with the associated measurement uncertainty (a 95% confidence level). (**d**) Exponential curves obtained using the best-fit curve of the measured values of *α* for both porcine and kidney tissues.

In Figure 9, the values of the thermal properties of porcine and bovine kidney tissue, measured at different temperatures in steady-state heat transfer conditions, are compared. Figure 9a shows the bar plot reporting the average values of *k* attained at temperatures of 23.2–23.9 °C, 35.4–36.9 °C, 40.9–41.5 °C, 46.2–48.8 °C, 56.4–56.7 °C, 69.5–70.1 °C, 75.8–76.4 °C, and 90.0–92.8 °C for both porcine and bovine samples, along with the associated measurement uncertainty (estimated with a 95% confidence level). It is possible to notice that, for instance, at room temperature, i.e., 23.2–23.9 °C, the difference between the average values of *k* associated with porcine kidney tissue and those related to bovine tissue was 4%. Likewise, at the highest investigated temperature (90.0–92.8 °C), the difference in terms of the average values *k* between the porcine and bovine tissue was <9%. Overall, at the same tissue temperature, the results of the *k* of porcine tissue were not statistically significantly different from the values of *k* measured for the bovine kidney (*p*-value > 0.1807). This was observed for all the investigated temperatures. Figure 9b depicts the curves interpolating the experimental *k* data for both porcine and bovine kidney samples. For the sake of comparison between the values of *α* measured for porcine and bovine tissues, the bar plots showing the average values attained at different increasing temperatures are presented in Figure 9c. As it was observed for *k*, considering the same tissue temperature, no statistically significant difference was found between the values of *α* measured for porcine tissue and the values recovered for bovine tissue (*p*-value > 0.1390). The exponential curves which best fit the measured values of *α* are shown in Figure 9d for both tissue types.

## 4. Discussion

The objective of the present experimental study was threefold. We devised the characterization of the thermal properties of ex vivo porcine and bovine kidney tissues from room up to supraphysiological (i.e., hyperthermic and ablative) temperatures in order to provide quantitative values of these properties in a wider thermal interval, compared to previous investigations that were limited to 45 °C [35]. Furthermore, with the aim of modeling the temperature-dependent variation of thermal properties of renal tissues, we evaluated the best-fit curves interpolating the empirical results. Lastly, we compared the thermal properties attained from the measurement of porcine and bovine tissues at different selected temperatures to also investigate inter-species dependence.

The measurement of the thermal properties, i.e., *k* and *α*, of biological media was performed in a temperature-controlled environment by means of a dual-needle probe, capable of measuring tissue temperature and delivering a specific thermal energy to the sample. The probe was connected to a commercial thermal property analyzer designed in accordance with ISO 2008 standards and in compliance with ASTM 5334 and IEEE 442 [61]. The same experimental technique has been employed and validated for the evaluation of the temperature-dependent properties of other biological tissues, e.g., porcine and bovine liver, porcine pancreas, brain, lung, heart, as well as ovine kidney tissues [7,12,33,37,40,62]. Moreover, the accurate reading capability of the used measurement system was tested, in our study, through the measurement of the thermal properties of a polymeric reference material. A difference lower than <1.5% compared with the results provided by the manufacturer on the same standard material was assessed, thus validating our measurement system.

For values of *k*, our measurements are in agreement with values registered at room, body, and hyperthermic temperatures for ex vivo porcine renal cortex, rabbit kidney, human renal pelvis, human renal medulla, and human renal cortex (Valvano et al.) using self-heating thermistors [35]. Table 5 reports the average values of *k* for different kidney tissues investigated in the present study and other studies that used various tissue temperatures. At room temperature, our measurements resulted in average values of 0.549 W/(m·K) for porcine kidney tissue at 23.9 °C and 0.528 W/(m·K) for bovine tissue at 23.2 °C, whilst the values measured by Valvano et al. (at 23 °C) were 0.524, 0.525, 0.524, 0.525, and 0.529 W/(m·K) for ex vivo porcine renal cortex, rabbit kidney, human renal pelvis, human renal medulla, and human renal cortex, respectively [35]. Thus, at room temperature, the maximum percentage difference between our experimental results and the values reported by Valvano et al. is <5%. Likewise, at body temperature, our average values of *k* were 0.559 W/(m·K) (at 35.4 °C, porcine kidney) and 0.551 W/(m·K) (at 36.9 °C, bovine tissue), showing a maximum percentage difference of lower than 4% compared to the values reported at 37 °C by Valvano et al. in porcine renal cortex, rabbit kidney, human renal pelvis, human renal medulla, and human renal cortex. Concerning higher temperatures, i.e., close to 45 °C, we attained average values of *k* = 0.573 W/(m·K) (at 46.2 °C, porcine kidney) and *k* = 0.541 W/(m·K) (at 48.8 °C, bovine kidney). Hence, in this case, the maximum difference between the values reported by Valvano et al. (45 °C) [35] and our average results is lower than 5% (Table 5). Compared with the results reported by Valvano et al. [35], we further expanded the thermal range in which the thermal properties were assessed up to 90.0–92.8 °C to also cover hyperthermic and ablative temperature intervals. The attained values of *k* remained rather constant until the onset of water vaporization, i.e., close to 90 °C [9]. For both the *k* values of porcine and bovine tissues, the best-fit regression models interpolating experimental data were represented by exponential curves. Concerning porcine kidney tissue, at 92.8 °C, a 1.2-fold increase in the average value of *k* compared to its baseline value at room temperature was observed. Similarly, for bovine kidney tissue, at 90.0 °C, the average value of *k* was 1.3 times higher with respect to its value at nominal conditions. These results are in line with the experimental observations reported for ovine kidney tissues by Silva et al. [40]. Indeed, the maximum difference between the average values resulting from our analysis at 90.0–92.8 °C and the values reported for ovine kidney tissue at around 93 °C is <5% (Table 5). Interestingly, we observed, at the same tissue temperature, no statistically significant difference between the values of *k* measured for the porcine kidney and the values attained for bovine kidney tissue.

Concerning the analysis of the temperature dependence of the *α* of porcine and bovine kidney tissues, as a function of temperature (i.e., from 23.2–23.9 °C up to 90.0–92.8 °C), we noticed an exponential trend with increasing temperature, as also observed for the values of *k*. The obtained average values at room temperature were 0.155 mm^2^/s (at 23.9 °C) and 0.151 mm^2^/s (at 23.2 °C) for porcine and bovine tissues, accordingly. At room temperature, a maximum percentage difference of 14% was observed in comparison with the values measured by Valvano et al. (at 23 °C) in porcine renal cortex, rabbit kidney, human renal pelvis, human renal medulla, and human renal cortex using self-heating thermistor probes [35]. Moreover, a maximum difference of approximately 5% can be assessed by comparing our results and previous measurements of ovine kidney tissue [40] performed with the same dual-needle techniques employed in the present study. Table 6 shows the average values of *α* obtained in our investigation along with the results of other experimental trials performed on ex vivo ovine kidney, porcine renal cortex, rabbit kidney, human renal pelvis, human renal medulla, and human renal cortex [35,40]. As can be observed, the averages values of *α* attained in this study are slightly higher compared with the average values measured by Valvano et al. at room temperature, body temperature, and at 45 °C in porcine renal cortex, rabbit kidney, human renal pelvis, human renal medulla, and human renal cortex [35]. However, the mentioned results are still in accordance considering the evaluated measurement uncertainties, estimated with a 95% confidence level. As previously reported, exponential curves were selected as best-fit models interpolating the measured *α* values for both porcine and bovine kidney tissues. At the highest investigated temperatures, i.e., 90.0–92.8 °C, the average *α* values were subject to a 1.4- and 1.2-fold increase for porcine and bovine kidney tissues, correspondingly. Also in this case, the results are in accordance with the measurements reported in ovine kidney tissue [40], for which a ~1.3-fold increase in *α* at 93 °C was shown compared to its nominal value at room temperature (Table 6). Considering the same tissue temperatures for *α*, the differences between the evaluated thermal property of porcine and bovine renal tissue were not statically significant.

Overall, the *k* and *α* values of porcine and bovine tissues, evaluated in steady-state heat transfer conditions, appeared rather constant from room temperature up to the onset of water evaporation. Afterward, a more marked increase in thermal properties was assessed. This is in line with previous investigations on liver, heart, and lung tissues [12,33], in which the most substantial changes were observed at temperatures higher than 91 °C. From our results, at temperatures that are able to cause reversible and/or irreversible thermal damage in biological tissue, such as >43 °C and 60 °C [18], the thermal properties of porcine and bovine kidney tissues appeared rather comparable with those evaluated at room and body temperature. This aspect could be insightful from the perspective of optimizing minimally invasive thermal therapies since the prediction of the thermal outcomes in tissue could be facilitated by no substantial variations in thermal properties. However, at higher temperatures, i.e., approaching the water phase change, which can also be reached during thermo-ablative procedures, more prominent variations in thermal properties were observed. For the investigated thermal properties and both porcine and bovine kidney tissues, exponential fitting curves were selected to interpolate the measured values. This is in accordance with the trends of *k* and *α* as a function of temperature observed for other biological tissues, such as porcine and bovine liver tissues, as well as heart and lung tissues [12,33,37]. The exponential increase in terms of *k* and *α* with temperature could be attributed to tissue subcellular alterations, leading to variations in cellular and structural tissue levels. Indeed, the alteration of protein structures and protein denaturation due to supraphysiological temperatures and the modification of cell membranes can lead to the release of water from the tissue. Hence, these mechanisms, alongside the vaporization phenomenon and vapor diffusion and condensation, are capable of modifying the overall thermal behavior and thermal characteristics of biological media [9,34].

Concerning the comparison of the *k* and *α* at the same selected tissue temperatures (23.2–23.9 °C, 35.4–36.9 °C, 40.9–41.5 °C, 46.2–48.8 °C, 56.4–56.7 °C, 69.5–70.1 °C, 75.8–76.4 °C, and 90.0–92.8 °C), no statistically significant differences between porcine and bovine kidney tissue were reported, despite the anatomical and structural differences between the two tissue types. This observation may be insightful for the proper selection of the tissue models for the optimization of different procedures involving heat transfer in kidney tissue. Indeed, porcine and bovine kidney tissues have been advocated as experimental models for training and medical research, also involving the optimization of thermal therapies [23,47]. However, a thorough evaluation and comparison of the temperature dependence of their thermal features are lacking. The proposed comparison of the two tissues in terms of thermal properties evaluated in a broad temperature range may represent a step forward in the characterization of inter-species variabilities and the similarities of the thermal properties of kidney tissue.

In conclusion, this study presents the characterization of the thermal properties of porcine and bovine kidney tissues as a function of temperature, ranging from room up to supraphysiological thermal ranges. The provided quantitative values, as well as the best-fit regression models describing the trends of thermal properties, can be used as inputs for numerical models of thermal therapies. Indeed, mathematical equations concerning the trends of the thermal properties of kidney tissue as a function of temperature can be easily incorporated into in silico models for a more reliable description of the thermal behavior of biological tissue. Accurate information on the thermal behavior of kidneys is required to enhance the prediction capability of computational and mathematical tools toward more accurate therapy pre-planning. Moreover, the measured properties may be used for the realization of tissue-like materials for testing, validation, and the improvement of preclinical devices. Thus, the measured properties may be useful for the design of new investigations aiming to improve thermal-energy-based therapies such as catheter-based renal denervation and thermal ablation procedures for unhealthy tissue treatment. Furthermore, the inter-species variabilities analysis of the thermal properties of porcine and bovine tissue may provide insights into the refinement of protocols and choice of tissue models for bioheat transfer studies in experimental laboratories. Additionally, species-dependent specific data on the thermal properties of tissues can help realize more accurate simulation models with respect to the type of tissues chosen for experimental validation. The principal limitation of the present study is the use of ex vivo healthy animal tissue models. Thus, future in vivo studies should be conducted considering the influence of physiological phenomena such as metabolic heat and perfusion on the variance of thermal properties with temperature. In addition, in the present study, measurements were performed with the aim of preserving the structure of the kidneys and penetrating both the cortex and medulla with a dual-needle probe. Hence, the measurement of the thermal properties refers to the thermal characteristics of both these tissue structures, considered as a whole. It may be of interest, also considering possible implications for improving thermal ablation treatments, to evaluate, in future studies, possible variations in thermal properties for the different regions of kidney tissue. Furthermore, additional investigations should also be devised for the measurement of the *k* and *α* of human tissue as a function of temperature, considering both diseased and healthy kidney tissues for a thorough characterization of their thermal features.

## Figures and Tables

**Table 1:** Thermal conductivity *k* and thermal diffusivity *α* of ex vivo porcine kidney at different increasing temperatures. The average values and measurement uncertainty with a 95% confidence level are reported for the different tissue temperatures, registered by the SH-3 dual-needle sensor.

T [°C]	$k\;[\frac{W}{m\;K}]$		$\alpha \;[\frac{mm^{2}}{s}]$	
Mean	Mean	Uncertainty	Mean	Uncertainty
23.9	0.549	0.045	0.155	0.019
35.4	0.559	0.050	0.157	0.011
41.5	0.573	0.050	0.161	0.009
46.2	0.573	0.030	0.163	0.011
56.7	0.571	0.033	0.161	0.007
60.0	0.560	0.022	0.160	0.008
70.1	0.536	0.017	0.165	0.011
76.4	0.528	0.026	0.171	0.014
82.3	0.527	0.080	0.178	0.016
86.6	0.596	0.058	0.194	0.033
92.8	0.648	0.061	0.216	0.078

**Table 2:** Obtained values of regression coefficients of the exponential fitting model (Equation (6)) and the corresponding value of $R^{2}$ for both thermal conductivity *k* and thermal diffusivity *α* for the measurements performed on porcine kidney tissue.

Thermal Property	a	b	c	*R* ^2^
Thermal conductivity, *k* [W/(m·K)]	0.5559	4.096 × 10^−9^	0.1818	0.6824
Thermal diffusivity, *α* [mm^2^/s]	0.1582	1.218 × 10^−5^	0.09125	0.9875

**Table 3:** The thermal properties (*k* and *α*) of ex vivo bovine kidney at different increasing temperatures. The average values are reported alongside the measurement uncertainty (estimated with a 95% confidence level) for different tissue temperatures.

T [°C]	$k\;[\frac{W}{m\;K}]$		$\alpha \;[\frac{mm^{2}}{s}]$	
Mean	Mean	Uncertainty	Mean	Uncertainty
23.2	0.528	0.066	0.151	0.020
30.2	0.548	0.030	0.153	0.030
36.9	0.551	0.091	0.157	0.035
40.9	0.528	0.180	0.157	0.032
48.8	0.541	0.129	0.160	0.043
56.4	0.562	0.167	0.155	0.015
69.5	0.595	0.183	0.162	0.029
75.8	0.590	0.164	0.169	0.031
81.1	0.550	0.191	0.160	0.009
90.0	0.703	0.226	0.183	0.006

**Table 4:** Regression coefficients of the exponential fitting model (Equation (6)) employed for mathematically fitting the curves, interpolating the experimentally attained values of thermal conductivity *k* and thermal diffusivity *α* of bovine kidney tissue, as well as corresponding values of $R^{2}$.

Thermal Property	a	b	c	*R* ^2^
Thermal conductivity, *k* [W/(m·K)]	0.5465	1.128 × 10^−6^	0.1309	0.8052
Thermal diffusivity, *α* [mm^2^/s]	0.1544	6.031 × 10^−5^	0.06728	0.8094

**Table 5:** Thermal conductivity *k* as a function of temperature attained in this study for porcine and bovine kidney and in other studies on ovine kidney, porcine renal cortex, rabbit kidney, human renal pelvis, human renal medulla, and human renal cortex.

	Ovine Kidney [40]	Porcine Renal Cortex [35]	Rabbit Kidney [35]	Human Renal Pelvis [35]	Human Renal Medulla [35]	Human Renal Cortex [35]	Porcine Kidney (Present Study)	Bovine Kidney (Present Study)
T [°C]	Thermal Conductivity *k* [W/(m∙K)]							
3	-	0.500	0.499	0.485	0.503	0.503	-	-
10	-	0.509	0.508	0.499	0.510	0.512	-	-
17	-	0.517	0.517	0.512	0.518	0.521	-	-
20	0.520	-	-	-	-	-	-	-
23	-	0.524	0.525	0.524	0.525	0.529	-	0.528
24	-	-	-	-	-	-	0.549	-
30	-	0.532	0.535	0.537	0.532	0.538	-	0.548
32	0.520	-	-	-	-	-	-	-
35	-	-	-	-	-	-	0.559	-
37	-	0.540	0.544	0.551	0.540	0.547	-	0.551
41	-	-	-	-	-	-	-	0.528
42	-	-	-	-	-	-	0.573	-
45	-	0.550	0.555	0.566	0.549	0.557	-	-
46	0.530	-	-	-	-	-	0.573	-
49	-	-	-	-	-	-	-	0.541
56	-	-	-	-	-	-	-	0.562
57	-	-	-	-	-	-	0.571	-
60	-	-	-	-	-	-	0.560	-
63	0.510	-	-	-	-	-	-	-
70	-	-	-	-	-	-	0.536	0.595
76	-	-	-	-	-	-	0.528	0.590
80	0.530	-	-	-	-	-	-	-
81	-	-	-	-	-	-	-	0.550
82	-	-	-	-	-	-	0.527	-
87	-	-	-	-	-	-	0.596	-
90	0.600	-	-	-	-	-	-	0.703
93	0.670	-	-	-	-	-	0.648	-
94	0.700	-	-	-	-	-	-	-
95	0.740	-	-	-	-	-	-	-

**Table 6:** Values of thermal diffusivity *α* at different tissue temperatures obtained in the present study for porcine and bovine kidney tissue and in other studies for ovine kidney, porcine renal cortex, rabbit kidney, human renal pelvis, human renal medulla, and human renal cortex.

	Ovine Kidney [40]	Porcine Renal Cortex [35]	Rabbit Kidney [35]	Human Renal Pelvis [35]	Human Renal Medulla [35]	Human Renal Cortex [35]	Porcine Kidney (Present Work)	Bovine Kidney (Present Work)
T [°C]	Thermal Diffusivity *α* [mm^2^/s]							
3	-	0.130	0.132	0.133	0.129	0.128	-	-
10	-	0.132	0.134	0.134	0.133	0.132	-	-
17	-	0.135	0.136	0.135	0.137	0.136	-	-
20	0.146	-	-	-	-	-	-	-
23	-	0.137	0.137	0.135	0.140	0.139	-	0.151
24	-	-	-	-	-	-	0.155	-
30	-	0.140	0.139	0.136	0.144	0.143	-	0.153
32	0.147	-	-	-	-	-	-	-
35	-	-	-	-	-	-	0.157	-
37	-	0.143	0.141	0.137	0.148	0.147	-	0.157
41	-	-	-	-	-	-	-	0.157
42	-	-	-	-	-	-	0.161	-
45	-	0.146	0.143	0.138	0.153	0.151	-	-
46	0.148	-	-	-	-	-	0.163	-
49	-	-	-	-	-	-	-	0.160
56	-	-	-	-	-	-	-	0.155
57	-	-	-	-	-	-	0.161	-
60	-	-	-	-	-	-	0.160	-
63	0.147	-	-	-	-	-	-	-
70	-	-	-	-	-	-	0.165	0.162
76	-	-	-	-	-	-	0.171	0.169
80	0.174	-	-	-	-	-	-	-
81	-	-	-	-	-	-	-	0.160
82	-	-	-	-	-	-	0.178	-
87	-	-	-	-	-	-	0.194	-
90	0.201	-	-	-	-	-	-	0.183
93	0.208	-	-	-	-	-	0.216	-
94	0.208	-	-	-	-	-	-	-
95	0.209	-	-	-	-	-	-	-

## Data Availability

Data are made available by the authors upon request.
